# Measurement of Stigma in Men Who Have Sex with Men in Hanoi, Vietnam: Assessment of a Homosexuality-Related Stigma Scale

**DOI:** 10.1155/2013/174506

**Published:** 2013-10-30

**Authors:** Huy Ha, Michael W. Ross, Jan M. H. Risser, Huong T. M. Nguyen

**Affiliations:** ^1^The Institute of Population, Health and Development, 18 Alley 132, Hoa Bang Street, Cau Giay District, Hanoi 10000, Vietnam; ^2^School of Public Health, The University of Texas, Texas, TX 77030, USA; ^3^The Center for Community Health Research and Development, Vietnam

## Abstract

*Objective*. To develop and assess a homosexuality-related stigma scale among men who have sex with men (MSM) in Hanoi, Vietnam. *Methods*. We conducted a cross-sectional study using respondent-driven sampling in Hanoi, Vietnam, in 2011. We used a cross-validation approach. Factor analysis was performed, and interitem correlation matrices were constructed to identify the latent factor structures, examine the goodness of fit, and assess convergent and discriminant validity of the determined scales. Internal consistency checks were performed in split samples and whole sample, and separately for each determined factor. *Results*. The findings were consistent in split samples. Three homosexuality-related stigma factors were identified: enacted homosexual stigma, perceived homosexual stigma, and internalized homosexual stigma. The fit indices of the confirmatory factor analysis in both split samples supported the hypothesized three-factor structures (in subsamples A and B: *χ* ^2^/degrees of freedom ratio = 1.77 and 1.59, nonnormed fit index = 0.92 and 0.94, comparative fit index = 0.93 and 0.95, and the root mean square of approximation = 0.06 and 0.05, resp.). The interitem correlation supported the convergent and discriminant validity of the scales. The reliability of the three scales indicated good consistency (Cronbach's alpha: 0.79–0.84) across split samples and for the whole data. *Conclusion*. Our scales have good psychometric properties for measuring homosexuality-related stigma. These comprehensive and practical tools are crucial not only to assess stigma against MSM and its consequence, but also to guide the development of interventions targeting MSM, as well as to evaluate the efficacy of existing stigma reduction efforts in Vietnam and other countries with similar settings.

## 1. Introduction

In Vietnam, despite prevention efforts and treatment programs to control the epidemic, HIV rates among men who have sex with men (MSM) are increasing [1–3]. HIV/AIDS related stigmas are known barriers to prevention, care, and treatment efforts [4–6]. They also influence testing and treatment behaviors by inhibiting actions that might lead to the disclosure of HIV infection status or HIV-related stigmatized behaviors [5]. While there have been attempts to address HIV/AIDS related stigmas, they continue to influence testing and treatment decisions and contribute to the perpetuation of the epidemic [6].

HIV/AIDS stigma refers to the discrimination and prejudice from others towards individuals infected with HIV or towards those who are vulnerable to HIV such as MSM, sexual workers, and injection drug users [6]. Since male-to-male sex is perpetuating the HIV epidemic in Vietnam, it is important to understand the influence of homosexual stigmas on the continuing epidemic. Although homosexual behavior itself has never been illegal in Vietnam, male-to-male sex is not socially acceptable, and MSM have faced high levels of stigma and discrimination [7, 8]. Most MSM in Vietnam try to keep their sexual identities and behaviors a secret. This secrecy results in decreased testing for HIV and other sexually transmitted infections (STIs) and failure to follow HIV/AIDS prevention practices [9, 10].

Recently, Earnshaw and Chaudoir [11] developed a practical and comprehensive HIV stigma framework that was extended on the basis of recent stigma theories [12] and Goffman's work [13] with an emphasis on the individual-level conceptualizations of stigma [11]. According to the framework, three stigma mechanisms: enacted, anticipated, and internalized stigmas, are manifested among those who are HIV infected and those who have certain risk behaviors related to HIV infection such as MSM, commercial sex workers, and injection drug users. Earnshaw and Chaudoir also indicated that the multiple-stigma measurement framework better reflects how stigma actually impacts individuals and is therefore more useful in predicting “important psychological, behavioral, and health outcomes” than a single-stigma mechanism framework [11]. Based on the framework and stigma theories [12], we developed three forms of homosexual stigma measurement scales adapted from previous studies conducted in China. Enacted homosexual stigma (experienced stigma) refers to the actual experience of prejudice and discrimination that occur to a man because of his homosexual activities. Perceived homosexual stigma refers to a man's perception of how others respond if they know about his same-sex behaviors [14]. Internalized homosexual stigma (self-stigma) often relates to experienced stigma [15] and refers to negative self-perception and beliefs of a man related to his same-sex behaviors that are the result of self-feelings of shame and self-fear of devalued societal attitudes [16, 17]. 

In previous studies, Neilands et al. developed and assessed enacted homosexual stigma and perceived homosexual stigma scales and found that they had acceptable to fairly poor reliability (Cronbach's alpha = 0.69 and =0.45, resp.) [18]. Afterward, Liu et al. [19] developed and adapted the perceived homosexual stigma and internalized homosexual stigma scales from the previous studies of [20, 21] and found that they had good reliabilities (Cronbach's alpha = 0.85 and 0.78, resp.). However, his study did not include enacted homosexual stigma, which is very common among MSM. Furthermore, although stigma reduction has been one of the key imperatives for success in HIV/AIDS prevention programs [6, 22], there have been no studies in Vietnam that examine homosexuality-related stigma measurement scales. In addition, while there are some current interventions among MSM in Vietnam that target stigma-reduction works on HIV prevention, care, and treatment [10], these require validated stigma measurement scales appropriate to the cultural context of Vietnam.

The low reliability of the enacted homosexual stigma scale, the inconsistent findings of perceived homosexual stigma scales in previous studies, and most importantly the need to account for differences in the manifestation of homosexuality-related stigmas in different cultural settings [6] suggest that further study is needed to refine the homosexual stigma structure. Therefore, in this paper, our purposes are to develop and assess homosexuality-related stigma measurement scales. The HIV-stigma scales we assess will serve as resources for researchers studying homosexuality stigma in Vietnam by offering a comprehensive set of homosexual stigma measures for use in the design and evaluation of interventions targeting homosexual stigma. 

## 2. Methods

### 2.1. Study Design and Subject

Participants were drawn from another larger cross-sectional study conducted in Hanoi from August 2010 to January 2011. Participants were men selected from the MSM community in Hanoi. To be eligible for the study a participant had to: (1) be a biological male; (2) be aged between 18 and 49 years; (3) have lived in Hanoi for at least 3 months prior to the survey; (4) report having sexual contact with other men at least twice during the 90 days prior to the study; (5) have a valid referral coupon; and (6) be willing and able to provide informed consent.

### 2.2. Recruitment and Data Collection

Respondent Driven Sampling (RDS) approach was used for participant enrollment. Ten eligible individuals (seeds) who had large social networks and were representatives of the MSM population in Hanoi were chosen. Each seed was given three uniquely coded coupons to recruit other eligible peers in his social network. After each eligible MSM was interviewed, he was given three unique coupons to recruit three additional eligible persons from his social network. The process was repeated until the desired sample size was reached and there were five to seven waves of RDS recruitment. The eligible and consenting MSM participants were interviewed at the counseling and testing clinic in the Hanoi Dermatology Hospital. The 30–45 minute face-to-face interviews were conducted using a structured questionnaire. An incentive equivalent to 10 USD was given to each participant after the interview and an additional incentive equivalent to 2.5 USD was provided for each successful recruitment of a friend. The study was approved by Johns Hopkins University's Center for Communication Programs, the University of Texas Health Science Center at Houston, and the Institutional Review Board (IRB) of the Vietnam Institute for Social Development Studies, which has registered with the National Institutes of Health (NIH; IRB00006556). The study was implemented by the Center for Community Health Research and Development (CCRD). All research staff were trained in research ethics and the protection of human subjects. Verbal consent was obtained for all survey interviews. All interviews were conducted in private settings to ensure confidentiality and protection of subjects' identity.

### 2.3. Measurement

A modified version of the 11-item perceived homosexual stigma scale, the 9-item enacted homosexual stigma scale, and the 8-item internalized homosexual stigma scale were adapted from previous studies [18–20, 23]. The questions measuring perceived homosexual stigma and internalized homosexual stigma were adapted from similar questions in the studies conducted by Liu et al. and Bruce [19, 20]. Due to the frequency in which stigma and discrimination occurring in health care settings is cited as a barrier to access to health facilities among MSM [6, 22], the following question was added to the stigma scale: “*Many health staffs often show unpleasantness or negative attitude while dealing with homosexual individual*”. Liu et al. [19] indicated that the scales measuring these two types of stigma exhibited good internal reliability (*α* = 0.85 and =0.78, resp.).

For the enacted stigma scale, we added two questions and modified six questions from the original scale of Neilands et al. [18]. The two added questions were “*how often have you been refused in receiving health care because of your homosexuality” *and *“how often are you afraid of seeking health care because of your homosexuality”*. The internal reliabilities of the original scale for this stigma type were moderate (*α* = 0.69) and good (*α* = 0.75) in the studies of Neilands et al. and Díaz et al., respectively [18, 23].

Four agreement/disagreement choices ranging from “*strongly agree*” to “*strongly disagree*” were designed for the questions about perceived and internalized homosexual stigma. In addition, we used a four-point Likert scale, ranging from “*never*” to “*many times*” for the questions about enacted homosexual stigma.

The questions were initially drafted in English and then translated into Vietnamese. Both English and Vietnamese versions were reviewed by two research staff members fluent in both English and Vietnamese. Content validity of modified questions was assessed by pilot testing the questionnaire during group discussions and individual interviews with 18 MSMs, to ensure accuracy of translation and clarity of modified questions (linguistic and cultural appropriateness). 

### 2.4. Data Analysis

We used a cross-validation method, in which the data sample was randomly split into two equal nonoverlapped subsamples (sub-sample A and sub-sample B): one was used for model development and the other was used to validate the model; then, the model and validation sets were crossed-over in successive rounds [24]. Exploratory factor analysis (EFA) was used for model development [25] and confirmatory factor analysis (CFA) [26] and inter-item correlation matrices [27] were used for testing model validation.

First, we empirically determined the number of latent factor structures underlying the study data by three rules: (1) the eigenvalue = one criterion; (2) the scree plot; and, (3) the proportion of variance extracted. For the first rule, factors with eigenvalues greater than one were considered significant for interpretation [28]. For the scree plot, we determined a “break” at the point where the curve first changes pitch. Only those factors appearing before the break were considered. For the third rule, the cumulative proportion of variance extracted by successive factors was considered to suggest the number of significant factors retained. After the number of latent factors was determined, Varimax rotation was performed to provide the relationship among individual items for each factor. Items with an absolute value loading of 0.30 or greater on one factor, and less than 0.30 on other factors were considered and retained for interpretation [29, 30].

Then, we used the interitem correlation analysis [27] and CFA [26] to examine the convergent and discriminant validities of the recommended scales. We constructed an inter-item correlation matrix, which is a table displaying the correlation of each item with every other item. The analysis of the inter-item correlation matrix is based on the principle that each item in the matrix should correlate more highly with other items of similar constructs (convergent validity) than items of different constructs (discriminant validity) [27]. For CFA, we used goodness-of-fit indices to assess overall fit of the expected model including the chi-square/degrees of freedom ratio (*χ* ^2^/df), the nonnormed fit index (NNFI), the comparative fit index (CFI), and the root mean square error of approximation (RMSEA) [31, 32]. The *χ* ^2^/df is measure of global fit. A value less than five indicated an adequate model fit while a value equal to or less than two indicated a good fit [33]. The RMSEA is a popular measure of fit and considered a standardized measure of error of approximation. In general, an RMSEA value of 0.05 or less indicates excellent model fit, and values between 0.06 and 0.10 show good fit [32]. NNFI, also called the Tucker-Lewis index, is one of the fit indices less affected by sample size and recommended for routine use [32]. This indicator varies from 0 to 1, a value of 0.95 or greater indicating very good fit. CFI is a measure of how much better the model fits than an independence model. This indicator is independent of sample size. A CFI value of 0.95 or higher indicates good fit [31, 32].

Internal consistency reliability checks were performed for sub-sample A, sub-sample B and the overall sample for the three scales and separately for each determined scale. Cronbach's alphas of ≥0.7-0.8 were considered to have good or excellent reliability [34]. Demographic findings, scale internal reliability values, EFA and inter-item correlation analysis were performed using Stata version 12.0 software. CFA was demonstrated using Lisrel Program version 8.80.

## 3. Results

A total of 451 men were eligible and recruited for the study. Most participants were young (mean age: 23 years old, standard deviation = 5, range 17–43 years old). The majority were studying or had completed college/university or higher (68%), while the remainder had completed secondary/high school (29%), or finished primary school (4%). The majority (94%) reported that they were single or had never married, while 6% stated that they were married, separated or divorced. Close to three-quarters of the men (68%) stated that they had been born outside Hanoi but lived in Hanoi, while the remainder had been born in Hanoi and lived in Hanoi (32%). More than a half of men (57.2%) in our study were students of colleges/universities. Surprisingly, nearly half of the men (48.9%) answered, “I am not sure about my gender,” when asked, “which of the following best characterizes your gender?”. Meanwhile, more than a quarter of the men (27%) answered, “I am a man,” 21% answered, “I am a woman,” and 4% answered, “I am transgender”.

The 28 adapted and modified items measuring the three factor constructs (enacted homosexual stigma, perceived homosexual stigma, and internalized stigma) are shown in Table 1.

**Figure 1:**
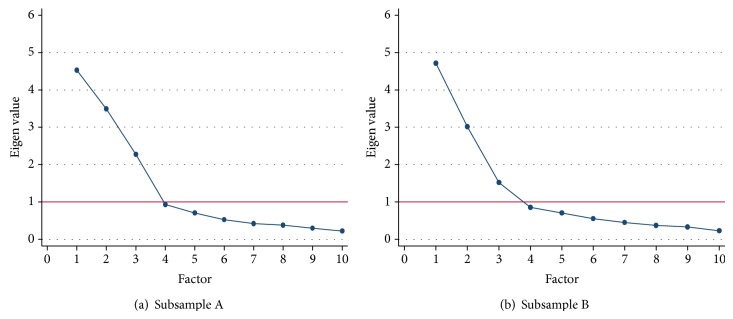
The first 10 factors.

The results of the EFA for both sub-samples A and B were similar. The eigenvalue = one rule showed three factors with eigenvalue greater than one that were retained (see Table 2). The remaining factors with eigenvalue less than one were removed. The scree plotting of the eigenvalues of the first 10 of 28 factors (Figure 1) also suggested retention of the first three factors. Similarly, the result of the “third rule” suggested the inclusion of factors 1, 2 and 3. The cumulative variances explained by the first three factors in sub-sample A and B were 82.8% and 83.3%, respectively. Only less than 17.2% of cumulative variance was explained by the other remaining factors.

Varimax-rotated factor loadings for 28 items for both sub-sample A and sub-sample B are presented in Table 3. The similar findings across split samples were found. A total of 7 items loaded on factor 1 measuring men's experiences of homosexual discrimination, which was labeled as enacted homosexual stigma. Eleven items loaded on factor 2 defining negative attitudes of the community toward stigmatized persons, which we named as perceived homosexual stigma. Eight items loaded on factor 3, which asked about the beliefs of participants about self-shame and fear leading to self-devaluation and internal conflict (internalized stigma). These items measured a latent factor which was labeled as internalized homosexual stigma. Two items (item 6, item 8) with value loadings less than 0.30 were removed (Table 3).

The inter-item correlation matrix showed a clear pattern indicating that the correlations among items measuring the same factor constructs (enacted stigma, perceived stigma, and internalized stigma) (mean *r* = 0.36) were significant and consistent. These were, on average, substantially higher than the correlations among items measuring different factors (mean *r* = 0.09) (Table 4). These findings support the convergent and discriminant validity of the scales.

CFA findings in both sub-sample A and sub-sample B were similar and demonstrated the good fit of the model (Table 4). The *χ* ^2^/df was 1.77 in sub-sample A and 1.59 in sub-sample B (≤2, =good fit); NNFI was 0.92 in sub-sample A and 0.94 in sub-sample B (>0.95, =excellent fit); CFI was 0.93 in sub-sample A and 0.95 in sup-sample B (≥0.95, =excellent fit); and RMSEA was 0.06 in sub-sample A and 0.05 in sub-sample A (≤0.05, =excellent fit).

Cronbach's coefficient alpha was used to assess the scale reliability. The internal reliability of entire scale was very good (*α* = 0.82). For individual scales (factors), there were no substantial differences on alpha estimated between sub-sample A, sub-sample B and whole sample, which indicated from good to very good internal reliability (*α* = 0.73–0.83). For instance, Cronbach's coefficient alpha for the whole sample were 0.82, 0.82, and 0.79 for enacted homosexual stigma, perceived homosexual stigma and internalized homosexual stigma, respectively. The correlation between enacted and internalized homosexual stigma, between enacted and perceived homosexual stigma, and between perceived and internalized stigma were 0.36, −0.14 and 0.14, respectively and each was statistically significant (Table 5).

## 4. Discussion

The findings of the study indicate that the homosexuality-related stigma scale demonstrate good construct validity with three scales measuring enacted homosexual stigma, internalized homosexual stigma, and perceived homosexual stigma. In addition, the scales demonstrated very good internal consistency. CFA and the inter-item correlation analysis validated the results of the exploratory method consistently and supported the selection of the hypothesized three-factor structure and the distinctness of the constructs measured by the scale. These scales, which were derived from the comprehensive framework [11] and theories of stigmatization [12], with an emphasis on individual-level dimension of stigma, assess a broad range of stigmatizing experiences, perceptions, beliefs, and attitudes of Vietnamese MSM about homosexuality-related stigma which provide useful data to inform, design, and evaluate stigma reduction programs.

In our study, the overall reliability and scale reliabilities were examined in split samples and in the whole sample. All were fairly high, including the enacted homosexual stigma (experienced stigma) scale which either was not included or exhibited fair low reliability coefficient in previous studies [18, 19]. This type of stigma is very common among MSM and is related to internalized homosexual stigma [7, 8]. These findings provide evidence that our adapted enacted homosexual stigma scale may be relevant to the homosexual stigma experiences of our participants and therefore more fully capture the broad range of Vietnamese gay men's experienced homosexual stigma. 

In this study, we used a cross-validation methods for data analysis to avoid overfitting and to provide a more accurate estimate for generation performance of model selection [35]. To assess the convergent and discriminant validity of the scales, we used the inter-item correlation analysis and CFA. CFA currently is the preferred method for assessing factor construct and item variance [36] because it can reproduce the original theoretical formulation of the multitrait-multimethod correlation matrix (MTMM) [36, 37]. The MTMM was developed in 1959 by Campbell and Fiske [38] and is considered to be one of the best available tests of validity [39]. In addition, CFA can determine whether the data fit with hypothesized models using goodness-of-fit indices [39] and can also provide the degree of convergent and discriminant validity through examination of the size of the item factor loadings, and factor covariances and correlations [40]. The consistent findings given from CFA in split samples and inter-item correlation matrix analysis, as well as the significant correlations among the three scales in our study, provided clear evidence of the convergent and discriminant validity of the scales. 

There are some potential limitations that should be acknowledged. First, our data may not be representative of all Vietnamese gay men although Hanoi is the second largest city in Vietnam. Therefore, a larger study covering more cities besides Hanoi may be needed. Second, although efforts were made to recruit a sample representing various social segments of Hanoi's MSM population, a large sample of these men were students. Last, due to fear and actual experiences of homosexuality-related stigma and discrimination, men who want to keep their sexual identities secret may not be willing to be recruited into the study. Therefore, further studies taking different approaches to recruitment may be needed in Hanoi.

Despite these limitations, our study has provided a useful tool to measure common forms of stigma against MSM and assess their consequences. Given that stigma reduction has been prioritized in existing HIV/AIDS prevention programs, this tool can be used as a practical instrument to evaluate the effectiveness of HIV prevention programs targeting MSM as well as a useful reference for future studies on homosexuality-related stigma in Vietnam and other countries with similar settings.

## Figures and Tables

**Table 1:** Questionnaire items measuring homosexuality-related stigma (*n* = 451).

Items	Item wordings	Mean^%^	SD^@^
	*Enacted homosexual stigma (factor 1) *	***1.59***	***0.43***
Item 1	How often have you lost a job or career opportunity due to your engaging in homosexual activities	1.22	0.05
Item 2	How often has your family rejected (separated, disregarded, etc.) you because of your homosexuality	1.59	0.84
Item 3	How often have you lost a friend because that person know you are engaging in homosexual sex	2.07	1.07
Item 4	How often have you been teased or cursed by others because your engaging in homosexual sex	2.32	1.18
Item 5	How often have you been hit or beaten up due to your homosexuality	1.30	0.74
Item 6	How often have you been kicked out of school for being homosexual	1.01	0.12
Item 7	How often have you changed accommodation due to your homosexuality	1.38	0.77
Item 8	How often have you been refused in receiving health care because of your homosexuality	1.03	0.28
Item 9	How often are you afraid of seeking health care because of your homosexuality	2.38	1.19

	*Perceived homosexual stigma (factor 2) *	***2.28***	***0.49***
Item 10	Many people unwilling accept homosexual individuals	2.05	0.72
Item 11	Gay individuals would lose their heterosexual partners once they knew their homosexual relationship	1.87	0.76
Item 12	Many employers would underestimate a man due to his homosexuality regardless of his qualifications for the job	2.37	0.85
Item 13	Many people would treat a gay individual differently than others	2.05	0.80
Item 14	Many people have negative attitudes towards gay men	2.02	0.88
Item 15	Many people do not see gay individuals as normal men	2.13	0.85
Item 16	Gay individuals are not welcome in public gatherings, for example, party, night club, meeting, etc.	2.79	0.81
Item 17	Many families would be disappointed to have a gay son	1.89	0.84
Item 18	Many people think that most gay individuals are HIV positive and will die of AIDS	2.90	0.77
Item 19	Many people believe that gay individuals are promiscuous	2.40	0.83
Item 20	Many health staffs often show unpleasantness or negative attitude while dealing with homosexual individual	2.60	0.80

	*Internalized homosexual stigma (factor 3) *	***2.19***	***0.51***
Item 21	Sometimes, you wish you were not gay	2.10	0.80
Item 22	Sometimes, you think that if you were not homosexual, you would probably be happier	2.22	0.81
Item 23	To avoid disclosure of your homosexual status, you have tried to stop being attracted to men	2.27	0.73
Item 24	Sometimes, you wish you could become more sexually attracted to women	2.32	0.80
Item 25	You feel that being gay is a personal shortcoming for you	2.42	0.74
Item 26	Sometimes, you feel ashamed of your sexual orientation	2.40	0.97
Item 27	You are afraid family and friends will find out about your sexual orientation	1.81	0.77
Item 28	You try to look masculine in order to avoid other's rejection	1.96	0.74

Note: ^%^range (1–4); ^@^SD: standard deviation.

**Table 2:** Eigenvalues and variance explained by first three factors in the subsample A and subsample B.

Factor	Subsample A (*n* = 225)			Subsample B (*n* = 226)		
	Eigenvalues	% Variance	% Cumulative	Eigenvalues	% Variance	% Cumulative
I	4.53	36.4	36.4	4.71	42.4	42.4
II	3.49	28.1	64.4	3.01	27.1	69.6
III	2.72	18.3	82.8	1.52	13.7	83.3

**Table 3:** Items and corresponding factor loadings from the rotated factor structure matrix: principal axis factoring with a Varimax rotation.

Item	Subsample A (*n* = 225)			Sub-sample B (*n* = 226)		
	Factor 1	Factor2	Factor 3	Factor 1	Factor 2	Factor 3
	*Enacted homosexual stigma *					
Item 1	0.02	**0.41** ∗	−0.15	−0.10	**0.48** ∗	0.21
Item 2	−0.01	**0.62** ∗	0.10	0.05	**0.53** ∗	0.04
Item 3	−0.06	**0.79** ∗	−0.01	−0.06	**0.83** ∗	0.06
Item 4	−0.14	**0.89** ∗	0.11	0.01	**0.84** ∗	−0.02
Item 5	−0.00	**0.51** ∗	0.11	−0.04	**0.42** ∗	−0.12
Item 6	−0.09	0.21	0.13	−0.04	0.20	−0.05
Item 7	−0.08	**0.61** ∗	−0.06	0.05	**0.49** ∗	0.01
Item 8	−0.08	0.13	−0.08	0.05	0.28	0.27
Item 9	−0.11	**0.72** ∗	0.00	−0.08	**0.70** ∗	0.02

	*Perceived homosexual stigma *					
Item 10	**0.37** ∗	0.10	0.08	**0.55** ∗	0.01	0.10
Item 11	**0.38** ∗	0.04	0.11	**0.48** ∗	−0.06	0.16
Item 12	**0.51** ∗	−0.05	0.08	**0.57** ∗	0.11	0.12
Item 13	**0.74** ∗	−0.04	0.13	**0.71** ∗	−0.03	0.13
Item 14	**0.75** ∗	−0.11	0.06	**0.65** ∗	−0.05	0.21
Item 15	**0.74** ∗	−0.14	0.12	**0.70** ∗	−0.06	0.14
Item 16	**0.57** ∗	−0.03	0.12	**0.56** ∗	−0.08	0.02
Item 17	**0.62** ∗	−0.07	0.16	**0.74** ∗	0.01	0.13
Item 18	**0.37** ∗	−0.09	−0.02	**0.35** ∗	−0.02	0.09
Item 19	**0.47** ∗	−0.12	0.07	**0.34** ∗	−0.13	0.09
Item 20	**0.40** ∗	−0.08	−0.14	**0.46** ∗	−0.02	0.06

	*Internalized homosexual stigma *					
Item 21	0.15	−0.10	**0.70** ∗	0.13	−0.03	**0.69** ∗
Item 22	0.09	−0.16	**0.70** ∗	0.16	−0.05	**0.64** ∗
Item 23	0.13	0.12	**0.60** ∗	0.26	−0.05	**0.35** ∗
Item 24	−0.04	0.16	**0.59** ∗	0.01	0.09	**0.51** ∗
Item 25	−0.03	−0.01	**0.73** ∗	0.20	0.06	**0.54** ∗
Item 26	0.10	0.01	**0.70** ∗	0.12	−0.05	**0.43** ∗
Item 27	0.10	−0.01	**0.30** ∗	0.26	0.03	**0.32** ∗
Item 28	0.15	0.13	**0.58** ∗	0.28	0.01	**0.40** ∗

Note: ∗loadings ≥ 0.30.

**Table 4:** Correlation between the items of the scales in overall sample (*n* = 451).

Item no.	Enacted homosexual stigma							Perceived homosexual stigma											Internalized homosexual stigma							
	1	2	3	4	5	7	9	10	11	12	13	14	15	16	17	18	19	20	21	22	23	24	25	26	27	28
1	1.00																									
2	**.15**	1.00																								
3	**.39**	**.45**	1.00																							
4	**.29**	**.56**	**.73**	1.00																						
5	**.23**	**.41**	**.26**	**.51**	1.00																					
7	**.38**	**.38**	**.46**	**.57**	**.23**	1.00																				
9	**.17**	**.41**	**.64**	**.66**	**.34**	**.47**	1.00																			
10	−.02	.07	.05	.04	.06	.03	−.01	1.00																		
11	−.06	.04	.00	−.04	.00	−.01	.00	**.28**	1.00																	
12	.02	−.04	−.13	−.13	−.02	−.12	−.06	**.29**	**.28**	1.00																
13	−.05	.01	−.07	−.11	−.03	−.08	−.12	**.28**	**.27**	**.35**	1.00															
14	−.09	−.09	−.12	−.16	−.10	−.12	−.15	**.18**	**.21**	**.30**	**.66**	1.00														
15	−.10	−.08	−.14	−.19	−.11	−.15	−.20	**.23**	**.28**	**.38**	**.62**	**.66**	1.00													
16	.05	.01	−.13	−.12	.02	−.08	−.07	**.23**	**.21**	**.41**	**.40**	**.36**	**.38**	1.00												
17	.00	−.07	−.11	−.13	−.04	−.11	−.11	**.24**	**.27**	**.32**	**.44**	**.53**	**.53**	**.38**	1.00											
18	−.01	.01	−.08	−.17	−.11	−.14	−.09	.08	.06	**.18**	**.28**	**.19**	**.20**	**.34**	**0.20**	1.00										
19	−.08	−.14	−.06	−.16	−.08	−.10	−.05	.07	**.24**	**.14**	**.40**	**.32**	**.40**	**.25**	**0.29**	**0.38**	1.00									
20	.10	−.07	−.05	−.13	−.14	−.06	−.14	.11	.05	**.22**	**.25**	**.38**	**.25**	**.24**	**0.19**	**0.14**	**0.16**	1.00								
21	−.17	−.02	−.02	−.01	−.04	−.07	−.07	.06	.12	**.17**	**.25**	**.18**	**.24**	**.14**	**.16**	.03	**.18**	.00	1.00							
22	−.14	−.03	−.10	−.09	−.03	−.09	−.15	.08	.10	.13	**.16**	.08	**.15**	**.18**	**.19**	−.02	**.15**	−.05	**.64**	1.00						
23	−.03	.12	.04	**.16**	**.17**	−.07	.05	**.16**	.10	.12	.06	.10	**.16**	**.17**	**.24**	−.01	.08	−.03	**.38**	**.38**	1.00					
24	−.05	**.19**	**.19**	**.25**	.02	.04	.**14**	−.04	.02	−.05	.13	.04	.00	−.03	.06	.07	.05	−.13	**.39**	**.39**	**.33**	1.00				
25	−.15	.05	−.05	.08	.03	.00	.04	−.01	.08	.07	.08	.04	.11	.02	.07	.03	.06	−.12	**.51**	**.44**	**.38**	**.50**	1.00			
26	.01	.05	.02	.05	.13	−.03	−.04	.08	.11	.05	.18	.11	.14	.16	.15	−.01	.08	.01	**.53**	**.52**	**.45**	**.31**	**.56**	1.00		
27	.08	.00	−.06	−.06	.12	−.01	−.08	.08	.03	.02	.05	.11	.13	.13	.17	−.04	.04	.03	**.14**	**.19**	**.27**	**.16**	**.19**	**.26**	1.00	
28	−.04	.13	.08	**.16**	.13	.00	.04	**.19**	.09	**.15**	**.15**	**.15**	.11	**.22**	**.19**	.01	.03	−.03	.**34**	**.35**	**.47**	**.35**	**.44**	**.37**	**.23**	1.00

Note: correlations in bold: *P*-value < 0.05.

**Table 5:** Factor Cronbach's coefficient alpha and correlations of three homosexual stigma subscales.

Factors	Cronbach's coefficient alpha			Correlation	
	Sub-sample		Whole sample	1	2
	A	B			
(1) Enacted homosexual stigma *(7 items) *	0.83	0.80	0.82	1	
(2) Perceived homosexual stigma *(11 items) *	0.82	0.84	0.82	−0.14 (*P* = 0.05)	1
(3) Internalized homosexual stigma *(8 items) *	0.82	0.73	0.79	0.14 (*P* = 0.05)	0.36 (*P* < 0.05)
